# Investigating soil and ATCC bacterial strains for their ability to synthesize anisotropic gold nanoparticles

**DOI:** 10.1007/s00253-025-13689-7

**Published:** 2026-01-12

**Authors:** Islam M. Ahmady, Javad B. M. Parambath, Elsiddig A. E. Elsheikh, Gwangmin Kim, Changseok Han, Alejandro Pérez García, Ahmed A. Mohamed

**Affiliations:** 1 Department of Applied Biology, College of Sciences, University of Sharjah, Sharjah, 27272 United Arab Emirates; 2 Departamento de Microbiología, Universidad de Málaga, and Instituto de Hortofruticultura Subtropical y Mediterránea “La Mayora” (IHSM-UMA-CSIC), Málaga, 29071 Spain; 3 Center for Advanced Materials Research, Research Institute of Sciences and Engineering, University of Sharjah, Sharjah, 27272 United Arab Emirates; 4 Department of Civil and Environmental Engineering, Texas A&M University, College Station, TX 77843 USA; 5 Program in Environmental and Polymer Engineering, Graduate School of INHA University, 100 Inha-Ro, Michuhol-Gu, Incheon, 22212 Korea; 6 Department of Environmental Engineering, INHA University, 100 Inha-Ro, Michuhol-Gu, Incheon, 22212 Korea

**Keywords:** Anisotropic gold nanoparticles, Aryldiazonium gold(III) salt, Bacterial synthesis, Green

## Abstract

**Abstract:**

The current study investigated 17 bacterial strains for their ability to synthesize gold nanoparticles (AuNPs) from the aryldiazonium gold(III) salt (DS-AuCl_4_). The study aims to investigate the ability of bacterial cell biomass in the stationary phase of growth to synthesize AuNPs at 28 °C and 37 °C. Eleven bacterial strains were isolated from soil and identified using the VITEK® 2 system and 16S rRNA sequencing. An additional six strains were obtained from the American Type Culture Collection (ATCC). The investigated Gram-positive and Gram-negative bacterial strains successfully produced anisotropic AuNPs at a cell density of 2.0 McFarland (6.0 × 10^8^ CFU/mL). Nanoparticle formation was faster when samples were incubated at 37 °C than at 28 °C across all bacterial strains. The results of UV-vis spectroscopy confirmed the presence of AuNPs, with peaks observed centered at 550 nm. High-resolution transmission electron microscopy (HR-TEM) revealed a variety of morphologies, including spheres, rods, triangles, pentagons, hexagons, irregular shapes, and flower-like structures. Gram-positive and Gram-negative bacteria synthesized AuNPs of sizes 38.7 ± 26.0 and 34.0 ± 18.6 nm, respectively. Lattice-spacing analysis confirmed the formation of metallic AuNPs. Energy-dispersed X-ray spectroscopy (EDS) validated the presence of gold in the samples, and X-ray photoelectron spectroscopy (XPS) confirmed the elemental composition of AuNPs at 84.0 eV. These nanoparticles have potential applications in cancer therapy and diagnosis, antibacterial treatments, and drug delivery.

**Key points:**

• *The AuNPs were synthesized using various bacterial strains*

• *The gold precursor is aryldiazonium gold(III) salt*

• *Various anisotropic morphologies were obtained*

**Graphical abstract:**

Created in BioRender. Ahmady, I. (2025)

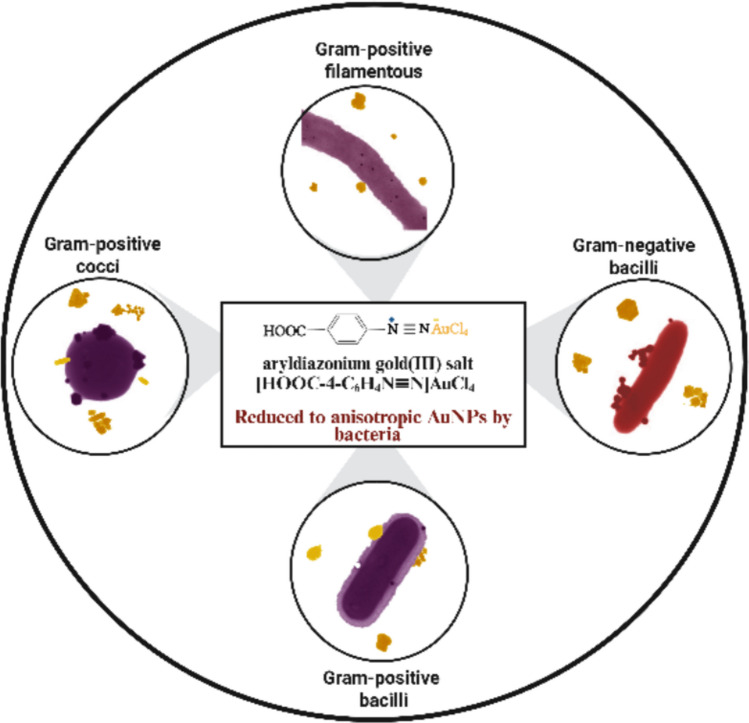

**Supplementary information:**

The online version contains supplementary material available at 10.1007/s00253-025-13689-7.

## Introduction

The reduction potential of bacteria plays a crucial role in nanoparticle formation. Microorganisms, such as bacteria, yeasts, and viruses, can produce metal and metal oxide nanoparticles by trapping metal ions outside or within microbial cells, enzymatic reduction, and capping. Metal ions are initially adsorbed onto the cell surface, followed by their reduction to NPs by enzymes produced by the microbes (Ghosh et al. 2021; Martinaga et al. 2024). The green synthesis of AuNPs using bacteria represents a significant advantage in nanotechnology for various applications (Schmitz et al. 2023). It is an eco-friendly and sustainable alternative to traditional chemical methods (Harish et al. 2022; Karnwal et al. 2024).

Many investigations have used HAuCl_4_ acid to synthesize AuNPs using organisms. Since the acid has a slightly higher reduction potential than many bacterial species, adjustments of pH, temperature, and time are required for efficient reduction. While some synthesis methods can be conducted under ambient conditions, others must be carried out for long hours or even days, using acidic or alkaline conditions, and run under H_2_ gas, with high gold(III) salt concentrations. For example, the cell-free supernatant of *Streptomyces albogriseolus* synthesized AuNPs when incubated with HAuCl_4_ at 37 °C in the dark for 24–96 h (El-Naggar et al. 2024). Konishi et al. (2007) study achieved AuNP deposition at 25 °C and pH 2.0–7.0 using the mesophilic Fe(III)-reducing bacterium *S. algae* with H_2_ gas as the electron donor. Ogi et al. (2010) synthesized spherical AuNPs and nanoplates at room temperature and pH 2.8 using cell extract from *S. algae* ATCC 51181 as both a reducing and shape-controlling agent. The extract reduced 1 mol/m^3^ aqueous HAuCl_4_ to elemental gold within 10 min when H₂ gas was provided as an electron donor. Du et al. (2007) obtained similar results when using *E. coli* DH5a. The AuNPs were formed when the mixture was kept under ambient conditions for 120 h. Cyanobacteria *Plectonemaboryanum* UTEX 485 reduced aqueous Na_3_[Au(S_2_O_3_)_2_] and HAuCl_4_ solutions at 25–100 °C for up to a month and at 200 °C for a day. Cyanobacteria reduced Na_3_[Au(S_2_O_3_)_2_] to AuNPs, accompanied by the formation of gold sulfide residues (Lengke et al. 2006).

Aryldiazonium gold(III) salts (DS-AuCl_4_) are preferred for AuNP synthesis due to their unique reactivity and functionalization capabilities (Ahmad et al. 2021; Parambath et al. 2023a, b). When reduced, these salts synthesize AuNPs under mild conditions, enabling precise control over size and morphology. Introducing aryldiazonium salts can add specific functional groups, enhancing their stability and preventing aggregation. DS-AuCl_4_ can operate spontaneously and effectively modify surfaces at a low reduction potential without the need for additional reducing chemicals. Our previous reports discussed the successful green synthesis of AuNPs from the exceptionally redox-active DS-AuCl_4_ using proteins and amino acids (Ahmady et al. 2019; Hameed et al. 2020a, b). DS-AuCl_4_ was used to fabricate nanostars (Hameed et al. 2022; Parambath et al. 2023a).

Currently, anisotropic gold nanoparticles have been utilized in various applications across different fields (Sajanlal et al. 2011; Ortiz-Castillo et al. 2020), such as the detection of breast cancer using surface-enhanced Raman spectroscopy (SERS) (Hameed et al. 2022), monitoring the organic pollutants using SERS (Parambath et al. 2023b), chemical catalysis (Priecel et al. 2016), biological and chemical sensing, catalysis, and medicine (Falahati et al. 2020), probing intracellular environments at the single-cell level (Austin et al. 2015), cancer drug delivery (Dreaden et al. 2012), cancer diagnostics and therapy (Austin et al. 2014), photothermal therapy for cancer (Sajanlal et al. 2011), detection of toxic ions and antibacterial agents (Ortiz-Castillo et al. 2020), and bioconjugation and labeling (Pissuwan et al. 2007).

In this study, 17 bacterial strains were investigated for their ability to reduce DS-AuCl_4_ to AuNPs. The bacteria are classified into two categories: soil-isolated bacteria and those from the American Type Culture Collection (ATCC). They were different in Gram staining and shape: Gram-positive filamentous, cocci, and bacilli, as well as Gram-negative bacilli. Eight Gram-positive bacteria, including *Streptomyces* sp. 11,* Streptomyces* sp. 28,* Streptomyces* sp. 36, *Dietzia* sp., *Enterococcus* sp.,* Enterococcus faecalis* ATCC 33186, *Staphylococcus aureus* ATCC 29213, and *Lactobacillus acidophilus* ATCC 4356, were used. Nine Gram-negative bacteria, including *Elizabethkingia* sp., *Pseudomonas* sp. 25, *Pseudomonas* sp. 26, *Pseudomonas* sp. 27, *Comamonas* sp., *Aeromonas* sp., *Shewanella algae* ATCC 51181, *Escherichia coli* ATCC 25922, and *Rhodopseudomonas palustris* ATCC 17007, were used. 

## Materials and methods

### Culture media

Nutrient agar and broth, Muller Hinton agar, starch nitrate casine (SNC) agar, *Pseudomonas* isolation agar (PIA), International *Streptomyces* Project No. 1 (ISP Medium No. 1) agar, and Rogosa agar and broth were all obtained from HiMedia, India.

### Source of bacterial strains

#### Standard bacterial strains

Standard bacterial strains were obtained from the American Type Culture Collection (ATCC) as follows: Gram-positive, *S. aureus* ATCC 29213, *E. faecalis* ATCC 33186, *L. acidophilus* ATCC 4356; Gram-negative, *S. algae* ATCC 51181, *E. coli* ATCC 25922, and *R. palustris* ATCC 17007.

## Isolation of bacteria from soil

Soil samples were collected from Sharjah City, UAE, at a depth of 5–10 cm into sterile plastic containers. To isolate heterotrophic bacteria, 1 g of soil was diluted into 9 mL of sterile distilled water and incubated in a shaking water bath at 28 °C for 1 h. The soil suspension was allowed to settle, then serially diluted tenfold in sterile distilled water to 10^–6^. Then, 100 µL of supernatant was cultured on nutrient agar and PIA. Agar plates were incubated for 24–72 h at 28 °C, after which the bacteria were further isolated and purified for identification. To isolate *Streptomyces*, 1 g of soil was diluted in 9 mL of sterilized distilled water and then incubated in a shaking water bath at 50 °C for 1 h. Similar to the previous procedure, samples were serially diluted tenfold to 10^−6^, and 100 µL of supernatant was inoculated onto SNC agar. Agar plates were incubated for 5–7 days at 28 °C. The *Streptomyces* colonies were further isolated and purified for identification. Purified bacteria were preserved in glycerol stock at −80 °C for further identification.

### Identification of bacterial strains

After purification, bacterial strains were identified based on their colonial morphology and Gram stain results.

### Screening of antibiotic production by *Streptomyces *using the disk diffusion method

Isolated *Streptomyces* strains were preliminarily screened for their ability to produce antibiotics and characterize their bioactive potential, before being selected for this study, which may relate to their ability to reduce DS-AuCl₄ and support future applications of the synthesized AuNPs. Eight different *Streptomyces* strains were isolated and tested for their ability to produce antibiotics using the disk diffusion method against two bacteria (*S. aureus* ATCC 29213 and *E. coli* ATCC 25922). The experiment followed previous protocols with modifications (Singh et al. 2016; Rammali et al. 2022) and adhered to the CLSI guidelines (Weinstein and Patel 2018). The *Streptomyces* bacteria were first grown on SNC agar for 1 week to allow antibiotic production in the medium. After that, antibacterial activity was evaluated using the agar disk diffusion method. Standard bacterial inocula were prepared from fresh 24-h culture plates using the direct colony suspension method. The optical density of the bacterial inoculum was adjusted to 0.5 McFarland standard at 600 nm using the DensiCHEK™ meter from bioMérieux. The inoculum was then evenly spread over the surface of Muller-Hinton agar using a cotton swab. Disks measuring 5 mm in diameter, made from SNC agar containing the mass of *Streptomyces* colonies and the antibiotics they produced, were placed onto the surface of the Muller-Hinton agar. Finally, the agar plates were incubated overnight at 37 °C.

### VITEK® 2 Compact microbial identification system

According to the manufacturer’s instructions, isolated bacteria were identified with the VITEK® 2 system. Bacteria were grown on nutrient agar and stained with Gram stain to determine the shape and Gram type. The optical density was adjusted to 0.46–0.62 McFarland using the DensiCHEK™ meter from bioMérieux, and a corresponding Gram-negative or Gram-positive kit from the same manufacturer was used to identify the bacterial strains.

### Identification of bacteria by 16S rRNA and Sanger sequencing

The three *Streptomyces* isolates with potential antibacterial activity were identified by 16S rRNA analysis, and some strains were further confirmed by 16S rRNA sequencing. A single colony from a fresh culture was grown overnight in nutrient broth. It was used to extract DNA according to the protocol for the BIOLINE ISOLATE II Genomic DNA kit. The concentration and purity of the DNA were measured using the NanoDrop and a 1% agarose gel. Then, Sanger sequencing was performed for the 16S rRNA region using the MicroSEQ Full Gene 16S rDNA Sequencing Kit from Thermo Fisher Scientific (catalog Number 4347484). This sequencing kit was used to identify *Streptomyces*. For the other bacterial strain confirmation, forward primer (515 F): 5′GTGCCAGCAGCCGCGGTAA3′ and reverse primer (1391 R): 5′GACGGGCGGTGTGTGCA3′ were used for partial amplification of the region, and PCR product amplification was confirmed on a 1.5% agarose gel. Afterward, capillary sequencing was performed using a Genetic Analyzer 3500 (Applied Biosystems, Thermo Fisher Scientific, USA). Chromas software and CAP3 tools were used for bioinformatic analysis and identification. Then, the Basic Local Alignment Search Tool (BLAST version BLASTN 2.17.0 +) was used to identify the strains (https://blast.ncbi.nlm.nih.gov/Blast.cgi; Zhang et al. 2000). The partial 16S rRNA gene sequences obtained from the isolates, along with their closest GenBank matches, were aligned using MEGA12 (Kumar et al. 2024). Five parallel computing threads were utilized. A neighbor-joining tree was generated (Saitou and Nei 1987), and its reliability was assessed with 1000 bootstrap replicates (Felsenstein 1985). Branches with more than 50% bootstrap replicates are removed, and bootstrap values are shown in the remaining nodes. The evolutionary distances were calculated using the maximum composite likelihood method (Tamura et al. 2004). The analysis included 22 nucleotide sequences, and pairwise deletion of ambiguous sites resulted in a final alignment of 1652 positions.

### Synthesis of aryldiazonium gold(III) salt

DS-AuCl₄ was synthesized following our literature procedure (Ahmad et al. 2019; 2020). Briefly, 4-aminobenzoic acid (0.411 g, 3.00 mmol) was dissolved in 6.00 M HCl (15.0 mL) and cooled to 0–5 °C in an ice bath. Separately, sodium nitrite (0.311 g, 4.50 mmol) was dissolved in deionized water (5.0 mL) and maintained at the same temperature. The NaNO_2_ solution was added dropwise to the acidic 4-aminobenzoic acid solution under vigorous stirring for 1 h, yielding the aryldiazonium intermediate. Subsequently, an aqueous solution of HAuCl_4_·3H_2_O (1.182 g, 3.00 mmol) in 10.0 mL of DI water was slowly added to the reaction mixture while maintaining ice-cooled conditions. Immediate precipitation of a canary yellow microcrystalline solid was observed. The product was collected by filtration, washed with cold deionized water (10.0 mL), air-dried, and stored at 4 °C for further use. A 1 mM solution of DS-AuCl_4_ was prepared and used for subsequent bacterial reduction experiments. Comprehensive characterization data, including UV-vis, FTIR, NMR, and XPS, have been published in earlier studies and are referenced in the cited references (Ahmad et al. 2019).

### Synthesis of AuNPs using bacteria

#### Preparation of bacterial inocula

To synthesize the AuNPs, a previously established protocol was followed (Ahmady et al. 2025). Bacterial strains were grown on nutrient agar and incubated aerobically; *E. faecalis*,* S. aureus*, and *E. coli* were incubated at 37 °C for 24 h. Soil-isolated bacteria, *S. algae* and *R. palustris*, were incubated at 28 °C for 24 to 72 h, depending on the strain. Then, fresh inocula were prepared in the nutrient broth and incubated under similar conditions. *L. acidophilus* was cultured in Rogosa medium (pH 6.2) and incubated anaerobically for 3 days at 35 °C. Fresh inoculum was prepared in Rogosa broth. *Streptomyces* spp. were cultured in SNC agar for 7 days at 28 °C aerobically, and then the broth inocula were prepared in ISP medium No. 1 broth. After that, all bacterial strain suspensions were centrifuged at 5000 rpm for 10 min, washed three times with DI water, and then resuspended in DI water at a cell density of 4.0 McFarland (12.0 × 10^8^ CFU/mL) for AuNP synthesis.

#### Synthesis of AuNPs using bacteria

To synthesize the AuNPs, 2 mL of bacteria was added to 2 mL of DS-AuCl_4_, resulting in a final cell density of 2.0 McFarland (6.0 × 10^8^ CFU/mL) and 0.5 mM, respectively. A control sample of DS-AuCl_4_ and DI water was prepared. The test tubes were incubated at 28 °C and 37 °C in the dark, under static aerobic conditions, and monitored for color changes. Except for *L. acidophilus*, tubes were incubated anaerobically under similar conditions. The tubes were inverted daily to mix the samples. Over time, the mixture’s color changed, gradually turning to shades of purple, and eventually purple precipitates formed, turning the aqueous solution colorless. Before measurements, the nanoparticle solution was shaken and sonicated for 5–10 s. The experiments were repeated three times for each bacterial strain to validate the results.

### Characterization of gold nanoparticles

#### High-resolution transmission electron microscopy (HR-TEM) with an energy dispersive spectrometer (EDS)

High-resolution transmission electron microscopy (JEOL JEM-2100, Japan) was employed to examine the size and morphology of the AuNP samples. An energy-dispersive spectrometer (EDS) equipped with the HR-TEM was used to identify the AuNPs on the bacterial samples. The samples were immobilized on 200 mesh copper TEM grids with a carbon support film (CF200-Cu-50, Electron Microscopy Sciences). A few drops of the sample solutions were placed on TEM grids and dried at room temperature. A negative staining method was utilized for the bacterial samples using UranyLess (Janecek and Kral 2016).

#### X-ray photoelectron spectroscopy characterization (XPS)

A Nexsa G2 surface analysis system from Thermo Fisher Scientific, UK, was used to examine the elemental composition and oxidation state of the sample. The instrument utilized monochromatized Al-Kα radiation (1486.6 eV) with a spot size of 400 μm and employed a flood gun for static charge compensation. Survey spectra were acquired at 200 eV, while high-resolution scans were conducted at 50 eV, all in an ultrahigh-vacuum environment at 10^−8^ mbar.

## Results

**Fig. 1:**
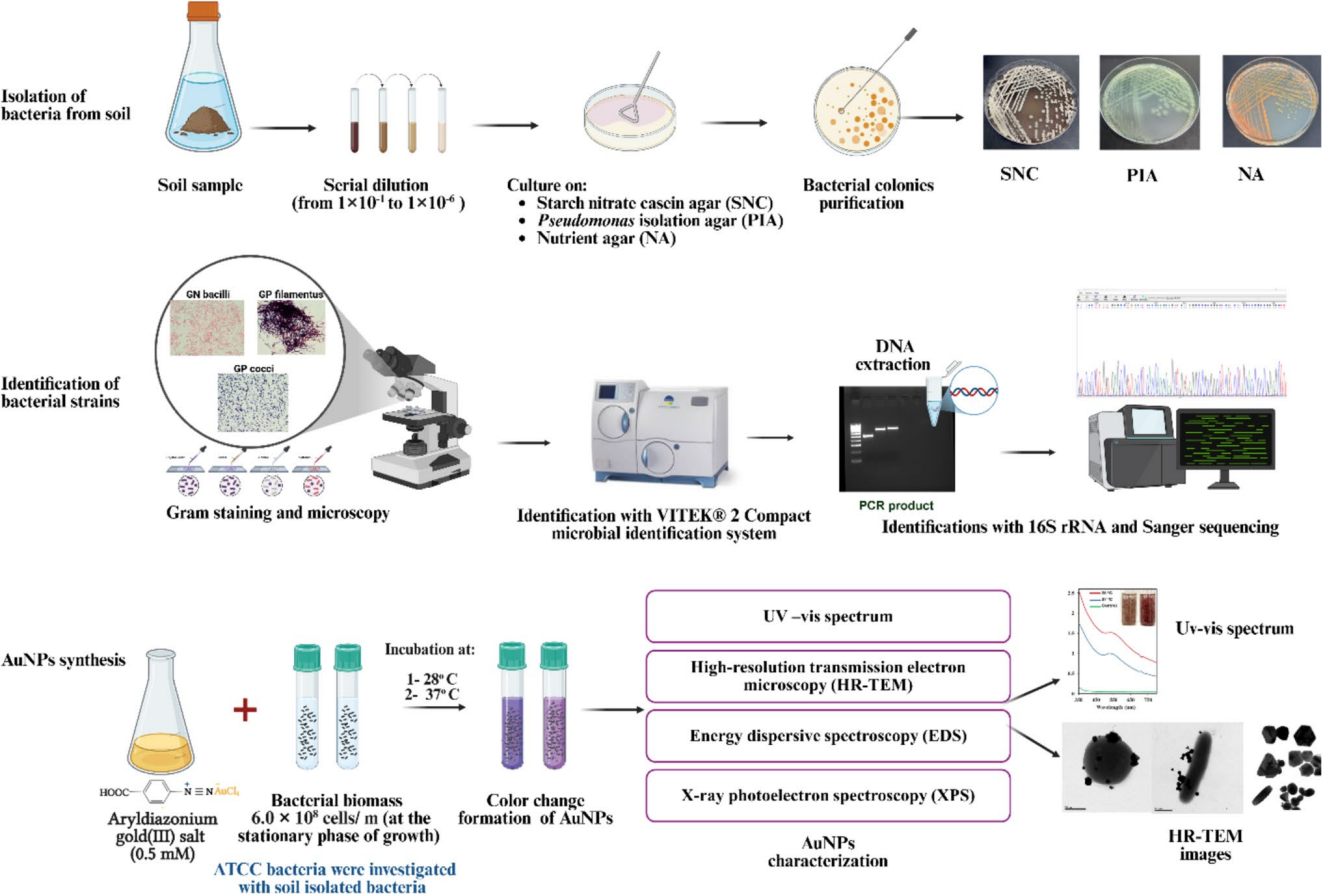
Isolation and identification of bacterial strains from soil and synthesis of AuNPs from DS-AuCl_4_. Step 1: isolation of bacteria using serial dilution of soil samples and culture on SNC, PIA, and nutrient agar. Step 2: identification of the isolated bacteria using Gram staining, VITEK 2, and 16S rRNA. Step 3: synthesis of AuNPs by adding 0.5 mM DS-AuCl_4_ and 6.0 × 10.^8^ CFU/mL of bacteria, and incubating at 28 °C and 37 °C. Characterize AuNPs using various techniques. Created using BioRender. Ahmady, I. (2025)

Figure 1 summarizes the steps and experimental procedures for bacterial isolation, identification, and AuNP synthesis.

### Identification of bacteria isolated from soil

**Fig. 2:**
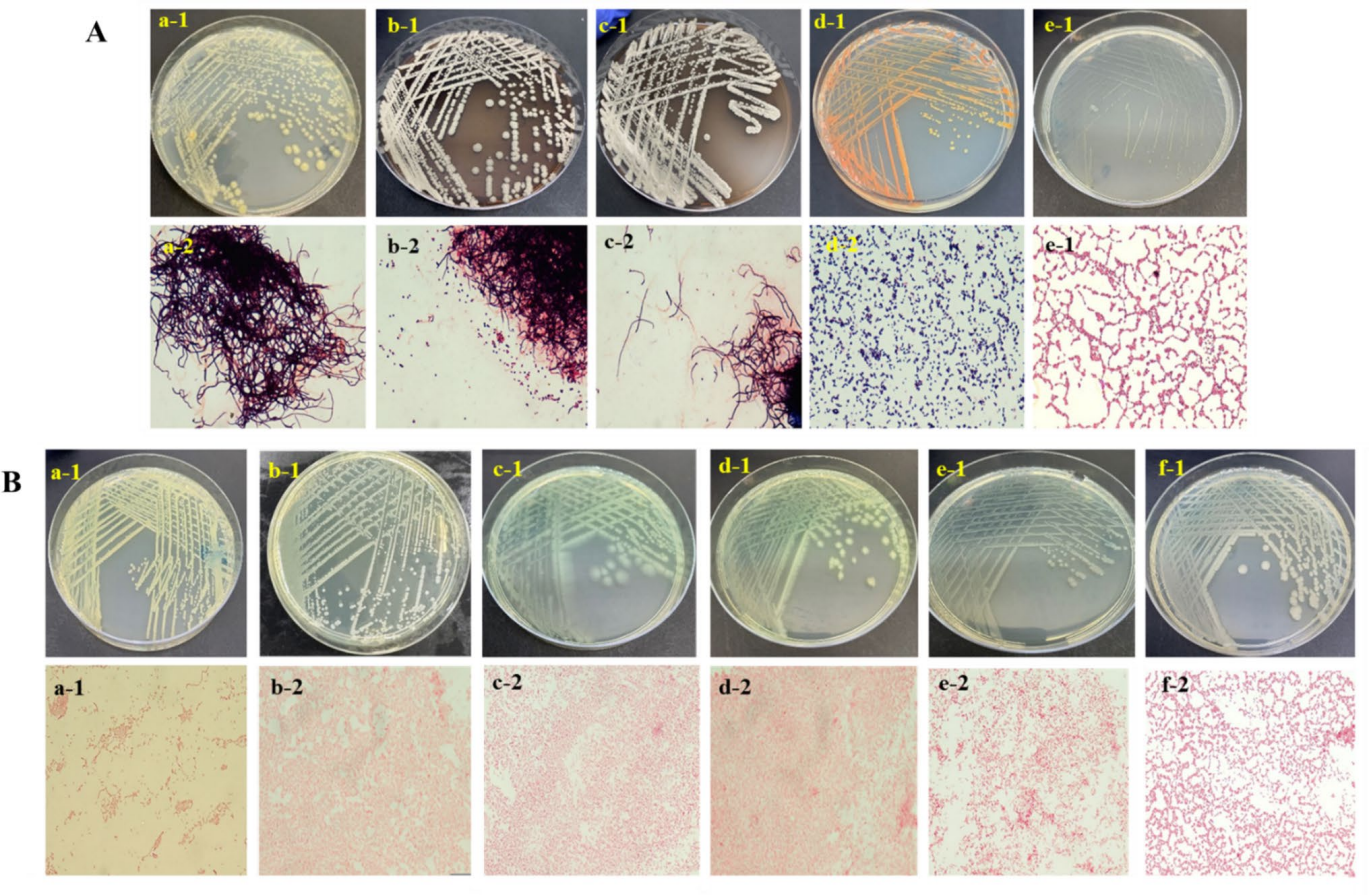
Images of bacterial strains isolated from soil. Culture on agar plates and Gram stain. **A** Gram-positive bacteria, (a1-2) strain no. 11 (*Streptomyces* sp.), (b1-2) strain no. 28 (*Streptomyces* sp.), (c1-2) strain no. 36 (*Streptomyces* sp.), (d1-2) strain no. 19 (*Dietzia* sp*.*)*,* and (e1-2) strain no. 35 (*Enterococcus* sp.). **B** Gram-negative bacteria, (a1-2) strain no. 24 (*Elizabethkingia *sp.), (b1-2) strain no. 25 (*Pseudomonas* sp.), (c1-2) strain no. 26 (*Pseudomonas* sp.), (d1-2) strain no. 27 (*Pseudomonas* sp.), (e1-2) strain no. 33 (*Comamonas* sp.), and (f1-2) strain no. 34 (*Aeromonas* sp.)

A total of 36 bacterial strains were isolated from soil, and 11 were identified and selected for the study, assigned the serial numbers 11, 19, 24, 25, 26, 27, 28, 33, 34, 35, and 36. Based on the colonial morphology and Gram stain, the selected bacteria were classified into five Gram-positive species: three filamentous and two cocci. Additionally, six Gram-negative rod bacteria were isolated. Figure 2 shows the images of culture plates and Gram staining results.

### Testing the antibiotic production by *Streptomyces *using disk diffusion

Eight *Streptomyces* spp. were isolated using SNC agar and differentiated based on colonial morphology and Gram staining. Three strains (*Streptomyces* spp. 11, 28, and 36) were selected for their ability to produce antibiotics against *S. aureus*, as indicated by the inhibition zones around the agar disks (Fig. S1; Table S1).

### VITEK® 2 Compact microbial identification system

The isolated strains were identified using the VITEK® 2 Compact microbial system. The Gram-positive coccus strain 35 was identified as *Enterococcus casseliflavus*. Additionally, the following Gram-negative bacilli bacteria were identified: strain 24 is *Elizabethkingia meningoseptica*, strain 25 is *Pseudomonas putida*, strains 26 and 27 show low discrimination between *Pseudomonas aeruginosa* and *Pseudomonas fluorescens*, strain 33 is *Comamonas testosteroni*, and strain 34 is *Aeromonas hydrophila* (Table 1; Fig. S2).

**Table 1:** Identification results of soil-isolated bacteria using the VITEK**®** 2 Compact identification system

Isolate ID	Isolate Source	Identification	Probability %	Confidence
19	Soil	Unidentified	-	-
24	Soil	*Elizabethkingia meningoseptica*	99%	Excellent identification
25	Soil	*Pseudomonas putida*	99%	Excellent identification
26	Soil	*Pseudomonas aeruginosa/Pseudomonas fluorescens*	-	Low-discrimination organism
27	Soil	*Pseudomonas aeruginosa/Pseudomonas fluorescens*	-	Low-discrimination organism
33	Soil	*Comamonas testosteroni*	99%	Excellent identification
34	Soil	*Aeromonas hydrophila*	99%	Excellent identification
35	Soil	*Enterococcus casseliflavus*	89%	Good identification

### Identification of bacteria using 16S rRNA and Sanger sequencing

The Gram-positive filamentous *Streptomyces* isolates were identified based on 16S rRNA gene sequencing. Strain 11 showed high similarity to *S. thermolilacinus*, while strains 28 and 36 were closely related to *S. althioticus* (Table 2). Gel electrophoresis images of genomic DNA and PCR products of these three strains are provided in the Supplementary Information (Fig. S3). Additionally, the Gram-positive coccus strain 19 showed a close match to *Dietzia maris*, and strains 26 and 27 were identified as closely related to *Pseudomonas aeruginosa* (Table 2). The phylogenetic tree and evolutionary relationships of the six sequenced strains, together with their closest GenBank matches, are shown in Fig. S4. The analysis also includes the five strains identified using the Vitek system, for which the corresponding reference sequences were retrieved from the GenBank database. *Desulfurococcus mobilis* archaeon was used as the outgroup.

**Table 2:** Identification of soil-isolated bacterial strains based on 16S rRNA gene sequencing

Isolate ID	Isolate Source	Closest match (Genus/Species)	Identity (%)	Accession number
11	Soil	*Streptomyces thermolilacinus* strain NIOT_MBCT17B	99.88	NR_125444.1
19	Soil	*Dietzia maris* strain 367–2	100	MT632637.1
26	Soil	*Pseudomonas aeruginosa strain DSM 50071*	99.73	NR_117678.1
27	Soil	*Pseudomonas aeruginosa strain* CUTMCTC09	99.86	PX506233.1
28	Soil	*Streptomyces althioticus* strain B 11	100	MK811432.1
36	Soil	*Streptomyces althioticus* strain NBRC 12740	100	NR_112254.1

### Synthesis of AuNPs with bacterial biomass

**Fig. 3:**
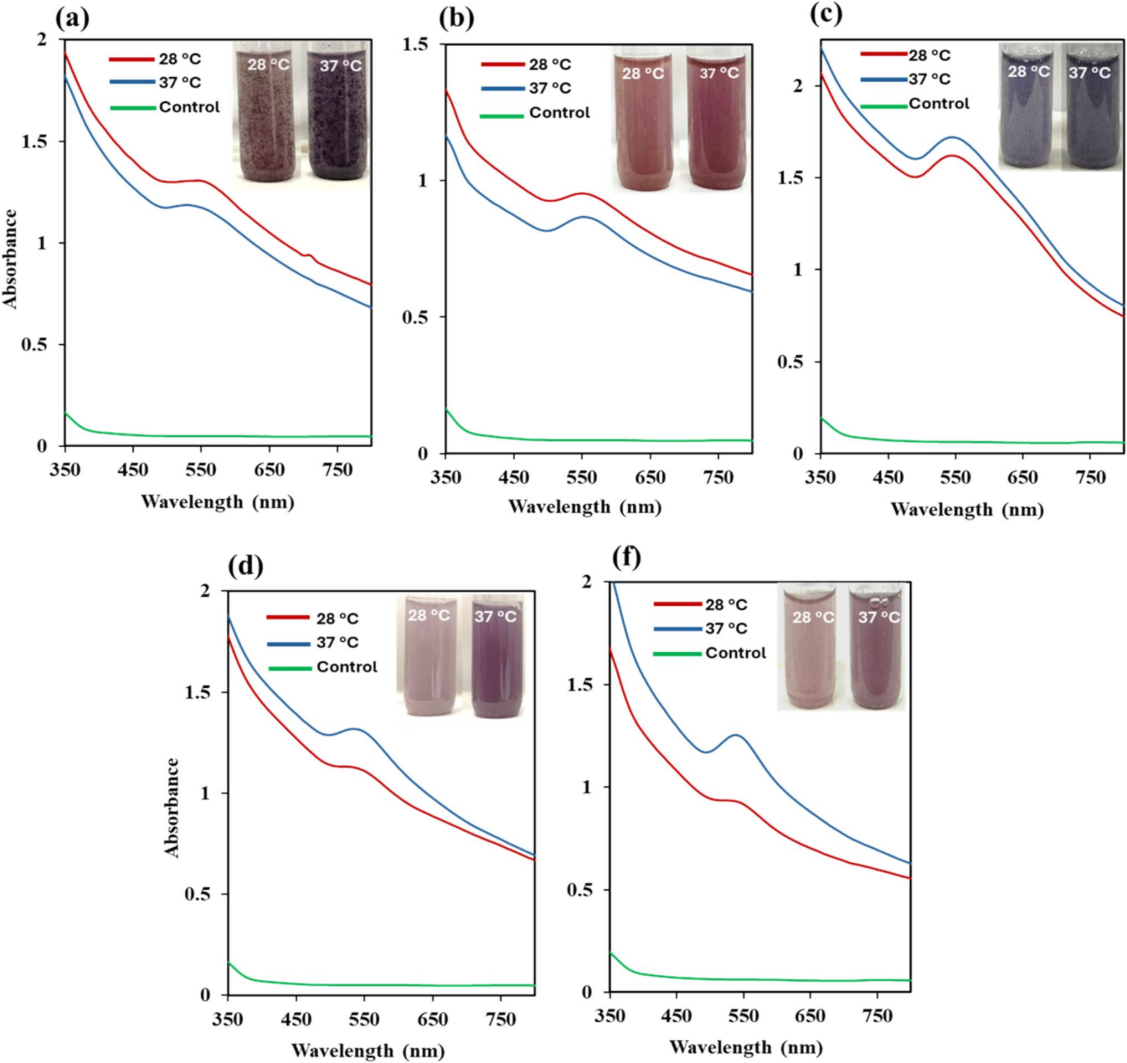
Representative UV–vis results of AuNPs synthesized from DS-AuCl_4_ by Gram-positive **a** *Streptomyces* sp. 36, **b** *Enterococcus* sp., and Gram-negative bacteria, **c** *Elizabethkingia* sp., **d** *Comamonas* sp., and **e** *S. algae* ATCC*.* Bacterial cell suspensions of 6.0 × 10^8^ CFU/mL were incubated with 0.5 mM DS-AuCl_4_ at 28 °C and 37 °C*.* Inset images of the test tubes displaying the development of color in the graph

All bacterial biomass of 6 × 10^8^ cells/mL in the stationary phase reduced 0.5 mM DS-AuCl_4_ and produced AuNPs, which was confirmed by the color change in the test tubes to a purple shade and the presence of the plasmon peak of the AuNPs in the range from 530 to 560 nm (Fig. 3, S6 and S7). No color change or plasmon peak was observed in the control sample of DS-AuCl_4_ and DI water. All investigated strains produced AuNPs within 24 h when incubated at 37 °C, except *Pseudomonas* sp. 25 and *R. palustris* ATCC 17007, which required 72 h. All bacterial strains required 48 to 120 h to form AuNPs when incubated at 28 °C, except *Dietzia* sp. and *Enterococcus* sp., which required 24 h (Table 3; Fig. S5). Our studies involved the stationary phase since the bacteria are metabolically active and reach their maximum growth stage and maintain a constant population before the reaction begins.

**Table 3:** Summary of the observations during the synthesis of AuNPs using 17 bacterial strains from DS-AuCl_4_ at the stationary growth phase

Source	Bacteria name	Bacteria Gram stain and shape	Reaction time		AuNPs Color	AuNPs shapes
			**28 °C**	**37 °C**		
Soil	*Streptomyces* sp. 11	GP^*^ filamentous	48 h	24 h	Bluish-Purple	Spherical, irregular
Soil	*Streptomyces* sp*.*28	GP filamentous	72 h	24 h	Maroon-Purple	Spherical, pentagonal, irregular
Soil	*Streptomyces* sp. 36	GP filamentous	72 h	24 h	Purple	Spherical, irregular
Soil	*Dietzia sp.*	GP cocci	24 h	24 h	Purple	Spherical, triangle, rods, irregular
Soil	*Enterococcus* sp.	GP cocci	24 h	24 h	Reddish-Maroon	Spherical, triangle, pentagonal, hexagonal, irregular
ATCC	*E. faecalis *(33186)	GP cocci	48 h	24 h	Purple	Spherical, triangle, irregular
ATCC	*S. aureus* (29213)	GP cocci	48 h	24 h	Purple	Spherical, cubical, irregular
ATCC	*L. acidophilus* (4356)	GP bacilli	120 h	24 h	Purple	Spherical, triangle, hexagonal, orchid-flower
Soil	*Elizabethkingia* sp.	GN^*^ bacilli	48 h	24 h	Purple-Blue	Spherical, triangle, pentagonal, hexagonal, rod, cubical, irregular
Soil	*Pseudomonas* sp. 25	GN bacilli	120 h	72 h	Purple	Stars, irregular
Soil	*Pseudomonas* sp. 26	GN bacilli	48 h	24 h	Purple	Spherical, irregular
Soil	*Pseudomonas* sp. 27	GN bacilli	48 h	24 h	Purple	Spherical, triangle, pentagonal, hexagonal, irregular
Soil	*Comamonas* sp.	GN bacilli	48 h	24 h	Purple	Spherical, triangle, pentagonal, hexagonal, cubical, rod, irregular
Soil	*Aeromonas* sp.	GN bacilli	96 h	24 h	Purple	Spherical, triangle, pentagonal, hexagonal, cubical, rod, star, irregular
ATCC	*S. algae* (51181)	GN bacilli	48 h	24 h	Purple	Spherical, triangles, irregular
ATCC	*E. coli* (25922)	GN bacilli	48 h	24 h	Purple	Spherical, triangle, pentagonal
ATCC	*R. palustris* (17007)	GN bacilli	96 h	72 h	Reddish-Maroon	Spherical, pentagonal

GP^*^ Gram-positive, GN^*^ Gram-negative

Most bacteria changed color in the test tubes, indicating the formation of AuNPs within 24 h at 37 °C. However, some bacteria required more than 24 h at lower temperatures for reduction to occur, suggesting that they produced AuNPs more rapidly when incubated at 37 °C than at 28 °C. UV-vis showed the plasmon peak of the AuNPs at wavelengths ranging from 530 to 560 nm (Fig. 3, S6, and S7). Moreover, the color darkens over time, indicating that the reduction persists for more than 24 h. The plasmon peaks were sharper at 37 °C across most strains compared to broad peaks at 28 °C (Fig. 3, S6, and S7). All Gram-positive bacteria demonstrated AuNP deposition after incubation under static conditions, suggesting that AuNPs were attracted to the bacterial cell wall, as HR-TEM confirmed. In contrast, most AuNPs were dispersed in the solution with Gram-negative bacteria. The developed colors were red, maroon, purple, and blue (Table 3; Fig. 3, S6, and S7). It has been observed that the bacteria produced more anisotropic shapes and created a dark purple color.

### Characterization of gold nanoparticles

#### High-resolution transmission electron microscopy and EDS analysis

**Fig. 4:**
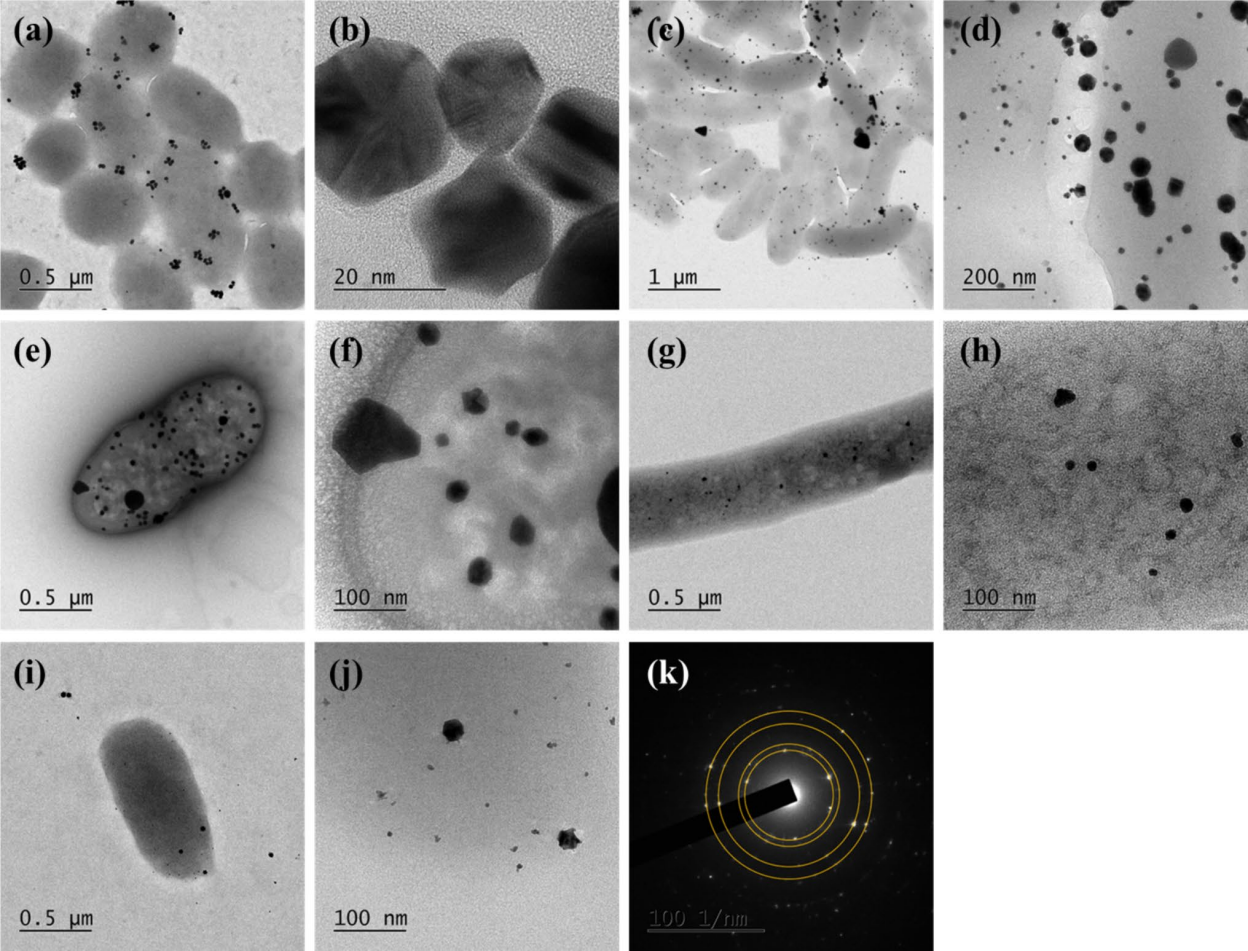
TEM images of synthesized AuNPs under different representative Gram-negative and Gram-positive bacteria. **a**–**b** *Elizabethkingia* sp., **c**–**d** *Comamonas* sp., **e**–**f** *Enterococcus* sp., **g**–**h** *Streptomyces* sp. 36, **i**–**j** *S. algae* ATCC, and **k** SAED pattern image of AuNPs

**Fig. 5:**
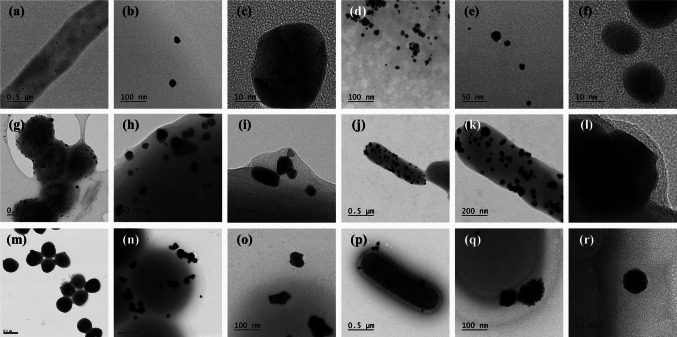
TEM images of the synthesized AuNPs under Gram-positive bacteria **a**–**c** *Streptomyces* sp. 11, **d**–**f** *Streptomyces* sp. 28, **g**–**i** *Dietzia* sp*.*, **j**–**l** *E. faecalis* ATCC, **m**–**o** *S. aureus* ATCC, and **p**–**r** *L. acidophilus* ATCC

**Fig. 6:**
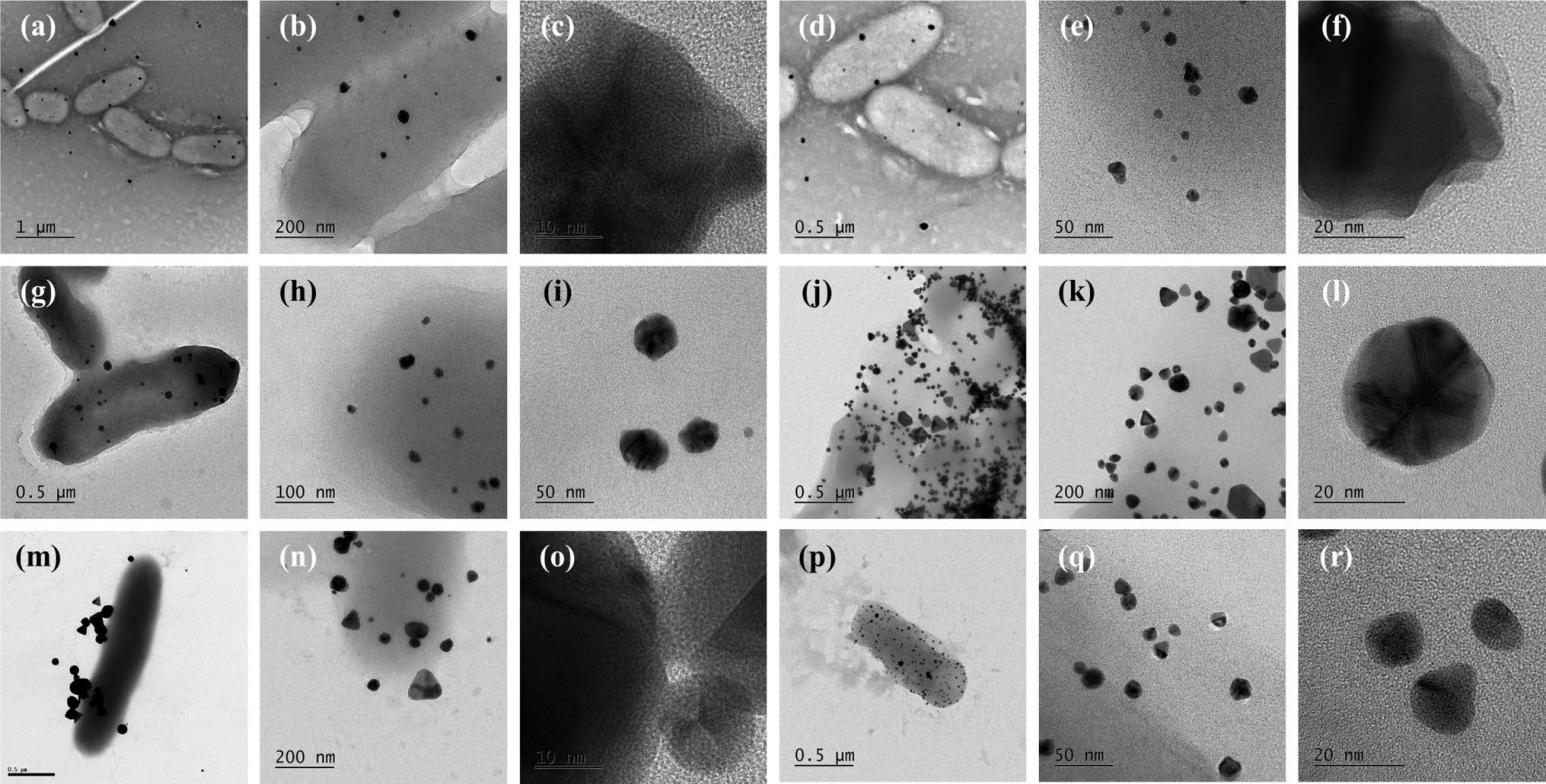
TEM images of synthesized AuNPs under Gram-negative bacteria **a**–**c** *Pseudomonas* sp. 26, **d**–**f** *Pseudomonas* sp. 27, **g**–**i** *Pseudomonas* sp. 25, **j**–**l** *Aeromonas* sp., **m**–**o** *E. coli* ATCC, and **p**–**r** *R. palustris* ATCC

Various morphologies were observed, including spherical, triangular, pentagonal, hexagonal, rod-like, orchid-like, and irregular shapes, as shown in Figs. 4, 5 and 6. Spherical and irregularly shaped morphologies were commonly synthesized for the Gram-positive bacteria (*Streptomyces* sp. 36 and *Enterococcus* sp.), as shown in Fig. 4. Triangular, pentagonal, and hexagonal-shaped nanoparticles dominated the sample of *Enterococcus* sp. bacteria. In the case of Gram-negative bacteria (*Elizabethkingia* sp., *Comamonas* sp., and *S. algae* ATCC 51181), spherical, triangular, and irregular morphologies were frequently observed, and pentagonal, hexagonal, cubical, and rod-shaped AuNPs were also synthesized with *Elizabethkingia* sp. and *Comamonas* sp. The shapes of AuNPs were highly diverse, whereas the bacteria produced mainly spherical particles. However, Figs. 5 and 6 show the typical shapes of AuNPs, including triangles, pentagons, hexagons, and cubes, formed by both Gram-positive and Gram-negative bacteria. The average particle sizes of the synthesized AuNPs using Gram-positive and Gram-negative bacteria were similar, which were 38.7 ± 26.0 and 34.0 ± 18.6 nm, respectively. Even though some particles larger than 100 nm were occasionally observed, the majority remained under 50 nm. Furthermore, EDS mapping images indicated that the bacteria successfully synthesized AuNPs (Fig. S8, S9, and S10).

### X-ray photoelectron spectroscopy characterization

**Fig. 7:**
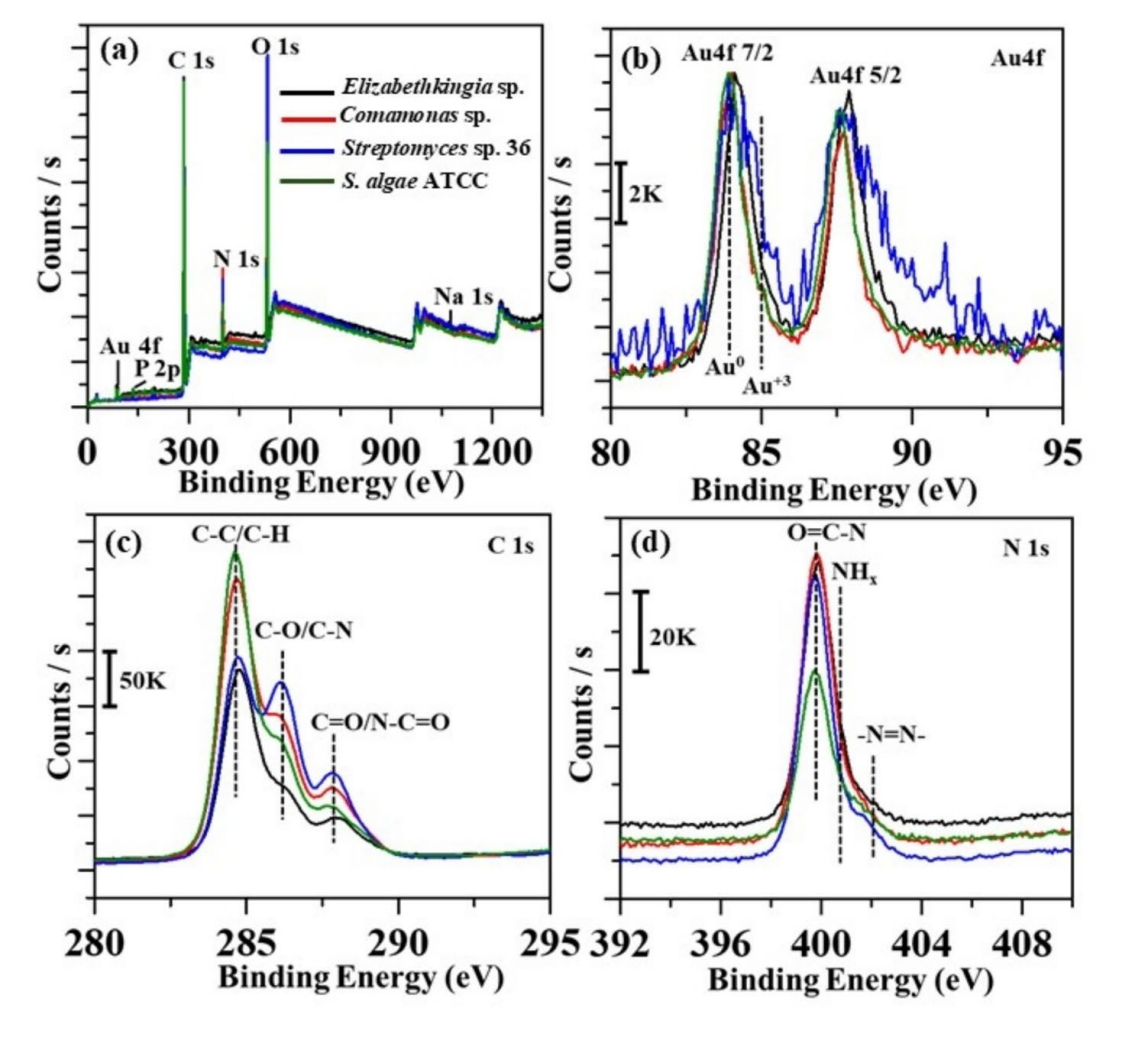
XPS analysis of AuNPs deposited using different bacterial species, *Streptomyces* sp. 36, *Elizabethkingia* sp., *Comamonas* sp., and *S. algae* ATCC. **a** survey spectra of AuNPs, **b** narrow scan of the Au 4f region, **c** narrow scan of the C 1 s region, and **d** narrow scan of the N 1 s region

The elemental composition of the AuNPs was analyzed using XPS. Figure 7a shows the survey spectra of AuNPs synthesized using *Streptomyces* sp. 36, *Elizabethkingia* sp., *Comamonas* sp., and *S. algae* ATCC. The inset highlights the narrow scan of the Au 4f region (Fig. 7b). In the survey spectrum, peaks for C 1 s, Au 4f doublet, N 1 s, and O 1 s are centered at 285, 84–88, 400, and 532 eV, respectively. All investigated samples primarily exhibit metallic gold, with the Au 4f7/2 peak centered at 84.0 eV and a spin-orbit coupling of 3.6 eV (Radnik et al. 2003; Almheiri et al. 2020), as shown in the inset. Notably, the *Streptomyces* sp. 36 sample exhibits residual diazonium salt due to partial reduction, as indicated by Au(III) peaks at 85.0 eV (Radnik et al. 2003; Almheiri et al. 2020). Further, analyzing the narrow scan shows the C 1 s region with three prominent peaks: C–C/C–H, C–O/C–N, and particularly C=O/N-C=O (Fig. 7c), which are associated with bacterial components, appearing at 284.7, 286.1, and 288.0 eV, respectively (Gam-Derouich et al. 2010). In the C 1 s region, the gold-bacterial bioconjugate shows a narrow peak with a shoulder at 286.1 eV from C–O bonds and a minor feature from C=O and N–C=O fragments (Fig. 7c). Interestingly, the strong COOH signal at 289.0 eV in the parent gold-aryl nanoparticles (Ahmad et al. 2019) is significantly reduced. Figure 7d displays a narrow scan of the N 1 s region, showing a sharp peak centered at 400.0 eV, attributed to peptide bonds in the bacterial components (Kjærvik et al. 2021). A slight shoulder at 401.8 eV, indicating an azo bond, is observed in sample *Streptomyces* sp. 36, suggesting an incomplete reduction of the diazonium salt under the given conditions. This feature may result from in situ X-ray-induced reduction during XPS analysis and is attributed to the −N=N– fragment (Toupin and Bélanger 2007).

## Discussion

It has been reported that bacterial cells and their extracts could synthesize AuNPs from HAuCl_4_. However, only a few studies reported the synthesis of AuNPs using bacterial biomass. To choose from the initial 36 isolates, we focused on strains that visibly reduced DS-AuCl_4_ and produced durable AuNPs. We also considered their well-known metabolic activities, such as metal transformation and AuNP formation. The selected strains include bacteria that play significant roles in the environment, particularly in bioremediation, such as *Pseudomonas* spp., *Dietzia* sp., and *Comamonas* sp., the metal-reducing *Shewanella algae*, antibiotic-producing *Streptomyces* spp., and photosynthetic *Rhodopseudomonas palustris*. Other strains, including *Enterococcus* sp*.*, *Enterococcus faecalis*, and *Lactobacillus acidophilus*, are lactic acid bacteria that are potential probiotics. Salt-tolerant *Staphylococcus aureus* and *Escherichia coli* are part of the human normal flora, while *Elizabethkingia* sp. and *Aeromonas* sp. are present in aquatic environments.

In this study, we successfully synthesized AuNPs using the 17 bacterial biomasses from DS-AuCl_4_. All experimental conditions remained constant, with no changes to pH (3.7), DS-AuCl_4_ concentration (0.5 mM), or bacterial concentration (6.0 × 10^8^ CFU/mL), as established in a previous study (Ahmady et al. 2025). The only variable was the incubation temperature, at 28 °C or 37 °C. Results showed that the formation of AuNPs was faster at 37 °C, and there was no noticeable difference in the ability of bacteria to synthesize AuNPs between Gram-positive and Gram-negative. Both could synthesize anisotropic AuNPs, and the formation of AuNPs did not affect bacterial morphology. TEM images showed that the filamentous, coccoid, and bacillary bacteria remained unchanged.

Although the study focuses on the ability of bacteria to synthesize AuNPs from DS-AuCl_4_, numerous anisotropic morphologies were observed, including spheres, rods, triangles, pentagons, hexagons, irregular shapes, and flower-like structures, depending on the bacterial strain. It is worth mentioning that the shapes of the AuNPs could not be directly linked to the Gram type of bacteria. For example, the Gram-positive bacterium *Streptomyces* sp. 11 exhibited fewer shapes, whereas *Enterococcus* sp. and *L. acidophilus* produced a greater variety of shapes. Among Gram-negative bacteria, *R. palustris* displayed fewer morphotypes, whereas *Elizabethkingia *sp. and *Aeromonas* sp*.* showed more diverse morphotypes (Table 3). The variation in shape among different bacterial strains may be linked to their cell wall structures, including peptidoglycan, specific enzymes, and metabolites produced by each species, such as reductase enzymes and extracellular polymeric substances (EPS), which could influence the shape and size of the synthesized AuNPs (Gholami-Shabani et al. 2015; Amina and Guo 2020; Ahmady et al. 2025). Previous studies have demonstrated that bacteria-secreted biomolecules can function as reducing and capping agents (Sathiyanarayanan et al. 2014; Dong et al. 2020). We hypothesize that variations in these biomolecules between strains contribute to the observed diversity in AuNP shapes. To further explore this hypothesis, future experiments will focus on identifying and characterizing these bacterial metabolites and enzymes to gain a deeper understanding of their role in shape control.

Differences in the cell envelope structures can explain the behavior of AuNPs toward Gram-positive and Gram-negative bacteria. Observations showed that AuNPs were attracted to Gram-positive bacteria more than to Gram-negative bacteria. This could be due to the fact that Gram-positive bacteria have a thick peptidoglycan layer enriched with teichoic acid, which contains numerous negatively charged sites. These sites facilitate strong adsorption and surface deposition of AuNPs (Caudill et al. 2020). In contrast, Gram-negative bacteria have an outer membrane rich in lipopolysaccharides that acts as both a physical and an electrostatic barrier, limiting nanoparticle attachment. As a result, AuNPs remain predominantly dispersed in solution rather than accumulating on the cell surface. This observation aligns with previous findings that show reduced nanoparticle binding to Gram-negative bacteria (Pajerski et al. 2019). These structural differences clearly explain why there is significant AuNP deposition on Gram-positive strains, while minimal interaction is seen with Gram-negative bacteria.

According to research by Lengke et al. (2006), the addition of [Au(S_2_O_3_)_2_]^3−^ caused the transformation of cyanobacteria filaments into their constituent cells. Then, membrane vesicles were released, forming irregular AuNPs around the cell (Lengke et al. 2006). In contrast, our results show that the formation of AuNPs by different bacteria with DS-AuCl_4_ did not significantly affect their morphology. The HR-TEM images showed that the filamentous, coccoid, and bacillus bacteria remained unchanged and that AuNPs formed around them. In the selected area, electron diffraction (SAED) analysis, the typical diffraction patterns of metallic gold were observed with a lattice spacing of 0.24, 0.20, 0.14, and 0.12 nm, corresponding to (111), (200), (220), and (311) planes, respectively (Das et al. 2010; Menon et al. 2017).

The observed XPS results suggest that there are variations in the strain’s metabolic capabilities or limited production of reducing metabolites, which are crucial for effective electron transfer during metal ion reduction. This observation underscores the importance of selecting microbial strains in biogenic synthesis. As bacterial components are rich in peptides, they exhibit an XPS survey spectrum similar to that of proteins with sharp C 1 s, O 1 s, and N 1 s peaks (Azioune et al. 2004). XPS analysis confirms the successful reduction of DS-AuCl_4_ in most bacterial samples, with metallic gold predominantly observed, as indicated by the Au 4f7/2 peak at 84.0 eV and a 4/3 intensity ratio between the Au 4f doublet peaks. However, in sample *Streptomyces* sp. 36, partial reduction of the DS-AuCl_4_ is evident, as shown by the presence of Au(III) peaks and residual azo bond signals. Notably, bare gold-aryl nanoparticles typically exhibit 0 at. wt% for N 1 s, but the pronounced N 1 s peaks observed in the bacterial bioconjugates are attributed to peptide linkages from bacterial proteins. This, along with strong C 1 s and O 1 s peaks, suggests that bacterial biomolecules, particularly peptides, play a vital role in the reduction process, facilitating the formation of AuNPs. Although bacterial synthesis effectively reduces gold ions, incomplete reductions highlight the need for optimized conditions to achieve complete conversion.

Here, we describe some studies involving HAuCl_4_, highlighting differences from DS-AuCl_4_. *Enterococcus* sp*.* (Gram-positive cocci) was isolated from the soil and used in the study. Oladipo et al. (2017) used a cell-free extract of *Enterococcus casseliflavus* W18 to reduce HAuCl_4_ under ambient conditions to produce spherical AuNPs with some degrees of polydispersity and aggregation, with a size of 8–42 nm in 30 min. *E. faecalis* ATCC 33186 (Gram-positive cocci) culture supernatant, when added to 20 mM HAuCl_4_ at 37 °C and pH 7.4, produced spherical AuNPs with a size range of 30–100 nm (Ashajyothi and Chandrakanth 2014). *L. acidophilus* ATCC 4356 (a Gram-positive bacillus), a cell-free extract of the probiotic strain *L. acidophilus*, was used by Abishad et al. (2024) to synthesize AuNPs at ambient temperature. The process involved a 1:4 ratio of 0.10 mM HAuCl_4_ and incubation at 37 °C for 60 min under constant stirring. The resulting AuNPs were uniform, spherical, and measured 8.55 ± 1.11 nm in diameter. In addition, *L. kimchicus* DCY51 cells synthesized AuNPs from HAuCl_4_ at 30 °C, yielding spherical particles with diameters of 5–30 nm (Markus et al. 2016). *Pseudomonas* spp*.* (Gram-negative bacilli) were isolated from the soil and studied. A few studies reported the extracellular biosynthesis of AuNPs using *Pseudomonas*. Synthesis of AuNPs by the supernatant of *P. aeruginosa* ATCC 90271, *P. aeruginosa* (2), and *P. aeruginosa* (1) was reported by Husseiny et al. (2007). Well-dispersed AuNPs in the 15–30 nm range were formed. Additionally, Rajasree and Suman (2012) reported the extracellular synthesis of AuNPs using *P. fluorescens*, producing nanoparticles with a size range of 50–70 nm using 10^−3^ M HAuCl_4_ after 24–48 h of shaking at 37 °C.

The color of the DS-AuCl_4_ is yellow when dissolved in water in the presence of bacteria. As bacterial metabolites reduced DS-AuCl_4_, the reaction color changed from yellow to pink, red, purple, and blue, depending on the bacterial species. The color change in the solutions indicates the synthesis of AuNPs. The color of the solution is associated with the AuNP morphology: anisotropic morphologies appear blue and dark red, and their plasmonic peaks shift to wavelengths above 550 nm. However, light colors indicate the spherical morphologies and are associated with a plasmonic peak characteristic of AuNP formation. In this research, the AuNPs are functionalized with carboxyl functional groups, which facilitate their binding to the bacterial cell wall. Our previous studies demonstrated the robust and highly dispersible properties of AuNPs synthesized using DS-AuCl_4_ (Ahmady et al. 2025). We observed high dispersibility of the aqueous solutions for all 17 bacterial species synthesized in this study.

The size of anisotropic AuNPs synthesized using bacteria varies significantly with reaction temperature, pH, the presence of live or dead bacteria, bacterial density, and bacterial strain. Recently, it has been demonstrated that the type of gold(III) salt plays a significant role in determining the rate of AuNP formation and the morphology, including spherical, rod-shaped, and nanostar shapes. Hence, in terms of sizes, anisotropic AuNPs can range from a few nanometers to over 100 nm. The synthesis can yield nanoparticles of varying sizes; thus, controlling reaction conditions is critical for producing uniform anisotropic AuNPs. In our study (Ahmady et al. 2025), *P. aeruginosa* produced average sizes of 26.0 ± 8.1 nm at 37 °C and 36.7 ± 7.7 nm at 42 °C using DS-AuCl_4_. *P. aeruginosa* synthesized 39.0 ± 9.1 nm at 25 °C and smaller sizes of 26 ± 8.1 nm at a higher temperature of 37 °C.

In this investigation, the reaction conditions included two temperatures: 28 °C and 37 °C; however, the pH and other reaction conditions remained constant. Bacterial cell suspensions of 6.0 × 10^8^ CFU/mL (constant in all reactions) were incubated with 0.5 mM DS-AuCl_4_ (constant in all reactions). The various bacterial strains formed different morphologies, but the average particle sizes of the synthesized AuNPs using Gram-positive and Gram-negative bacteria were similar: 38.7 ± 26.0 and 34.0 ± 18.6 nm, respectively. However, some particles were larger than 100 nm, while the majority remained under 50 nm.

To the best of our knowledge, no previous studies have examined the ability of *Dietzia* sp., *S. aureus* ATCC 29213 (Gram-positive cocci), *Elizabethkingia* sp., *Aeromonas* sp., and *Comamonas* sp. (Gram-negative bacilli) to synthesize AuNPs.

## Conclusion

The reduction potential of the gold(III) salts plays a crucial role in AuNP formation. DS-AuCl_4_ exhibited remarkable features, including operation at ambient conditions (28 °C and 37 °C), reduction at low concentration (0.5 mM), and time efficiency. This was achieved by using bacteria in the stationary phase of growth to demonstrate reduction capabilities across all types of bacteria, including Gram-positive and Gram-negative bacteria, bacilli, cocci, and filamentous bacteria. Moreover, this environmentally friendly process reduced the need for harsh chemicals and extreme conditions usually linked with such reactions.

A limitation of this study is the formation of various AuNP morphologies. This variability in shape can impede reproducibility. It is recommended that this issue be addressed in future work by fine-tuning the experimental conditions, which may include using shape-directing agents to control particle growth, increasing bacterial density, and adding salt to influence nucleation rates. Optimizing these parameters may enable the synthesis of monodisperse anisotropic AuNPs.

## Supplementary information

Below is the link to the electronic supplementary material.

ESM 1 (DOCX 5.33 MB)

## Data Availability

The authors declare that the data supporting the findings of this study are available within the paper and its Supplementary Information files. Should any raw data files be required in an alternative format, they are available from the corresponding author upon reasonable request.

## References

[CR1] Abishad P, Vergis J, Arya PR, Unni V, Vinod VK, Juliet S, Kurkure NV, Barbuddhe SB, Byrappa K, Rawool DB (2024) Facile one-pot synthesis of gold nanoparticles using *Lactobacillus acidophilus* as a potential photocatalytic agent against multi-drug-resistant pathogens of public health importance. Biomass Convers Biorefin 14:29787–29794

[CR2] Ahmad AAL, Panicker S, Chehimi MM, Monge M, Lopez-de-Luzuriaga JM, Mohamed AA, Bruce AE, Bruce MRM (2019) Synthesis of water-soluble gold–aryl nanoparticles with distinct catalytic performance in the reduction of 4-nitrophenol. Catal Sci Technol 9:6059–6071

[CR3] Ahmad AAL, Workie B, Mohamed AA (2020) Diazonium gold salts as novel surface modifiers: what have we learned so far? Surfaces 3:182–196

[CR4] Ahmad AAL, Marutheri Parambath JB, Postnikov PS, Guselnikova O, Chehimi MM, Bruce MRM, Bruce AE, Mohamed AA (2021) Conceptual developments of aryldiazonium salts as modifiers for gold colloids and surfaces. Langmuir 37:8897–8907 34291926 10.1021/acs.langmuir.1c00884

[CR5] Ahmady IM, Hameed MK, Almehdi AM, Arooj M, Workie B, Sahle-Demessie E, Han C, Mohamed AA (2019) Green and cytocompatible carboxyl modified gold–lysozyme nanoantibacterial for combating multidrug-resistant superbugs. Biomater Sci 7:5016–5026 31620700 10.1039/c9bm00935c

[CR6] Ahmady IM, Parambath JBM, Elsheikh EAE, Kim G, Han C, Pérez-García A, Mohamed AA (2025) Bacterial synthesis of anisotropic gold nanoparticles. Appl Microbiol Biotechnol 109:62 40064650 10.1007/s00253-025-13438-w PMC11893633

[CR7] Almheiri S, Ahmad AAL, Le Droumaguet B, Pires R, Mohamed AA, Chehimi MM (2020) Development of latent fingerprints via aryldiazonium tetrachloroaurate salts on copper surfaces: an XPS study. Langmuir 36:74–83 31786922 10.1021/acs.langmuir.9b03390

[CR8] Amina SJ, Guo B (2020) A review on the synthesis and functionalization of gold nanoparticles as a drug delivery vehicle. Int J Nanomedicine 15:9823–9857 33324054 10.2147/IJN.S279094 PMC7732174

[CR9] Ashajyothi C, Chandrakanth RK (2014) Biological synthesis and characterization of gold nanoparticles from *Enterococcus faecalis*. J Bionanosci 8:255–259

[CR10] Austin LA, Mackey MA, Dreaden EC, El-Sayed MA (2014) Optical, photothermal and surface chemical properties of gold and silver nanoparticles in biodiagnostics and therapy. Arch Toxicol 88:1391–1417 24894431 10.1007/s00204-014-1245-3 PMC4136654

[CR11] Austin LA, Kang B, El-Sayed MA (2015) Probing molecular cell event dynamics at the single-cell level with targeted plasmonic gold nanoparticles: a review. Nano Today 10:542–558

[CR12] Azioune A, Ben Slimane A, Ait Hamou L, Pleuvy A, Chehimi MM, Perruchot C, Armes SP (2004) Synthesis and characterization of active ester-functionalized polypyrrole–silica nanoparticles: covalent attachment of proteins. Langmuir 20:3350–3356 15875868 10.1021/la030407s

[CR13] Caudill ER, Hernandez RT, Johnson KP, O’Rourke JT, Zhu L, Haynes CL, Feng ZV, Pedersen JA (2020) Wall teichoic acids govern cationic gold nanoparticle interaction with Gram-positive bacteria. Chem Sci 11:4106–4118 34122876 10.1039/c9sc05436g PMC8152635

[CR14] Das SK, Das AR, Guha AK (2010) Microbial synthesis of multishaped gold nanostructures. Small 6:1012–1021 20376859 10.1002/smll.200902011

[CR15] Dong B, Liu G, Zhou J, Cai L, Wang J, Jin R (2020) Roles of molecular weight–fractionated extracellular polymeric substances in transformation of Au(III) to gold nanoparticles. Sci Total Environ 728:138889 32361363 10.1016/j.scitotenv.2020.138889

[CR16] Dreaden EC, Austin LA, Mackey MA, El-Sayed MA (2012) Size matters: gold nanoparticles in targeted cancer drug delivery. Ther Deliv 3:457–478 22834077 10.4155/tde.12.21 PMC3596176

[CR17] Du L, Jiang H, Liu X, Wang E (2007) Biosynthesis of gold nanoparticles assisted by *Escherichia coli* DH5α and direct electrochemistry of hemoglobin. Electrochem Commun 9:1165–1170

[CR18] El‑Naggar NE‑A, El‑Sawah AA, Elmansy MF, Elmessiry OT, El‑Saidi ME, El‑Sherbeny MK, Sarhan MT, Elhefnawy AA, Dalal SR (2024) Optimization of gold nanoparticle biosynthesis by Streptomyces albogriseolus using artificial neural networks. Sci Rep 14:4581 38403677 10.1038/s41598-024-54698-2 PMC10894868

[CR19] Falahati M et al. (2020) Gold nanomaterials as key suppliers in sensing, catalysis and medicine. Biochimica et Biophysica Acta – General Subjects 1864:129435. 10.1016/j.bbagen.2019.129435 31526869

[CR20] Felsenstein J (1985) Confidence limits on phylogenies: an approach using the bootstrap. Evolution 39:783–791 28561359 10.1111/j.1558-5646.1985.tb00420.x

[CR21] Gam-Derouich S, Carbonnier B, Turmine M, Lang P, Jouini M, Ben Hassen-Chehimi D, Chehimi MM (2010) Electrografted aryl diazonium initiators for photopolymerization. Langmuir 26:11830–11840 20568823 10.1021/la100880j

[CR22] Gholami-Shabani M et al (2015) Enzymatic synthesis of gold nanoparticles using sulfite reductase from *E. coli*. Process Biochem 50:1076–1085

[CR23] Ghosh S, Ahmad R, Banerjee K, AlAjmi MF, Rahman S (2021) Mechanistic aspects of microbe-mediated nanoparticle synthesis. Front Microbiol 12:638068 34025600 10.3389/fmicb.2021.638068 PMC8131684

[CR24] Hameed M et al (2020a) Protein-coated aryl modified gold nanoparticles for cellular uptake. Langmuir 36:11765–11775 32931295 10.1021/acs.langmuir.0c01443

[CR25] Hameed MK et al (2020b) Efficient synthesis of amino acid–capped gold nanoparticles. Amino Acids 52:941–953 32607864 10.1007/s00726-020-02862-z

[CR26] Hameed MK et al (2022) Arylated gold nanostars for SERS detection of breast cancer cells. Appl Surf Sci 583:152504

[CR27] Harish V et al (2022) Nanoparticle and nanostructure synthesis: controlled growth methods. Nanomaterials 12:3226 36145012 10.3390/nano12183226 PMC9503496

[CR28] Husseiny MI, El-Aziz MA, Badr Y, Mahmoud MA (2007) Biosynthesis of gold nanoparticles using *Pseudomonas aeruginosa*. Spectrochim Acta A Mol Biomol Spectrosc 67:1003–1006 17084659 10.1016/j.saa.2006.09.028

[CR29] Janecek M, Kral R (2016) Modern electron microscopy in physical and life sciences. InTech

[CR30] Karnwal A et al (2024) Gold nanoparticles in nanobiotechnology. ACS. Omega 9:29966–29982 10.1021/acsomega.3c10352 PMC11256298 39035946

[CR31] Kjærvik M et al (2021) Comparative NAP-XPS and cryo-XPS of bacterial cell envelopes. Front Chem 9:666161 34026730 10.3389/fchem.2021.666161 PMC8132101

[CR32] Konishi Y et al (2007) Microbial deposition of gold nanoparticles by Shewanella algae. Electrochim Acta 53:186–192

[CR33] Kumar S, Stecher G, Suleski M, Sanderford M, Sharma S, Tamura K (2024) MEGA12: molecular evolutionary genetics analysis for adaptive computing. Mol Biol Evol 41:1–9 10.1093/molbev/msae263 PMC11683415 39708372

[CR34] Lengke MF, Fleet ME, Southam G (2006) Morphology of gold nanoparticles synthesized by cyanobacteria. Langmuir 22:2780–2787 16519482 10.1021/la052652c

[CR35] Markus J et al (2016) Intracellular synthesis of gold nanoparticles by Lactobacillus kimchicus. Enzyme Microb Technol 95:85–93 27866630 10.1016/j.enzmictec.2016.08.018

[CR36] Martinaga L et al (2024) Applications of bacterial dehydrogenases and oxidases in gold nanoparticle synthesis. Appl Microbiol Biotechnol 108:62 38183486 10.1007/s00253-023-12853-1 PMC13497615

[CR37] Menon S, Rajeshkumar S, Venkat Kumar S (2017) Review on biogenic synthesis of gold nanoparticles. Resour-Eff Technol 3:516–527

[CR38] Ogi T et al (2010) Room-temperature synthesis of gold nanoparticles using *Shewanella algae* extract. J Nanopart Res 12:2531–2539

[CR39] Oladipo IC et al (2017) Enterococcus species for one-pot biofabrication of gold nanoparticles. J Photochem Photobiol B 173:250–257 28601037 10.1016/j.jphotobiol.2017.06.003

[CR40] Ortiz-Castillo JE, Gallo-Villanueva RC, Madou MJ, Perez-Gonzalez VH (2020) Anisotropic gold nanoparticles: recent synthetic methodologies. Coord Chem Rev 425:213489

[CR41] Pajerski W et al (2019) Attachment efficiency of gold nanoparticles by bacteria. J Nanopart Res 21:186

[CR42] Parambath JBM et al (2023a) Immobilization of gold–aryl nanoparticles on graphene oxide. J Cluster Sci 34:577–586

[CR43] Parambath JBM et al (2023b) SERS performance of cubic gold nanoparticles for environmental monitoring. Res Chem Intermed 49:1259–1271

[CR44] Pissuwan D et al (2007) Targeted destruction of macrophages using bioconjugated gold nanorods. J Nanopart Res 9:1109–1125

[CR45] Priecel P et al (2016) Anisotropic gold nanoparticles in catalysis. Chin J Catal 37:1619–1650

[CR46] Radnik J, Mohr C, Claus P (2003) Core-level binding energy shifts of supported gold nanoparticles. Phys Chem Chem Phys 5:172–177

[CR47] Rajasree SR, Suman T (2012) Extracellular biosynthesis of gold nanoparticles by *Pseudomonas fluorescens*. Asian Pac J Trop Dis 2:S796–S799

[CR48] Rammali S et al (2022) Antimicrobial activity of Streptomyces species from cold soils. Sci Rep 12:17233 36241756 10.1038/s41598-022-21644-z PMC9568536

[CR49] Saitou N, Nei M (1987) Neighbor-joining method for reconstructing phylogenetic trees. Mol Biol Evol 4:406–425 3447015 10.1093/oxfordjournals.molbev.a040454

[CR50] Sajanlal PR, Sreeprasad TS, Samal AK, Pradeep T (2011) Anisotropic nanomaterials: structure and growth. Nano Rev 2:5883 10.3402/nano.v2i0.5883 PMC3215190 22110867

[CR51] Sathiyanarayanan G et al (2014) Carbohydrate-polymer-encrusted gold nanoparticles via bacterial EPS. RSC Adv 4:22817–22827

[CR52] Schmitz FRW et al (2023) Colorimetric detection of *Pseudomonas aeruginosa* by aptamer-functionalized AuNPs. Appl Microbiol Biotechnol 107:71–80 36418544 10.1007/s00253-022-12283-5

[CR53] Singh V et al (2016) Isolation and screening of novel actinomycetes. Front Microbiol 7:1921 27999566 10.3389/fmicb.2016.01921 PMC5138215

[CR54] Tamura K, Nei M, Kumar S (2004) Prospects for large phylogenies using neighbor-joining. PNAS 101:11030–11035 15258291 10.1073/pnas.0404206101 PMC491989

[CR55] Toupin M, Bélanger D (2007) Thermal stability of aryl-modified carbon black. J Phys Chem C Nanomater Interfaces 111:5394–5401

[CR56] Weinstein MP, Patel JB (2018) Methods for dilution antimicrobial susceptibility tests for bacteria that grow aerobically, 11th ed. CLSI, Wayne, PA.

[CR57] Zhang Z, Schwartz S, Wagner L, Miller W (2000) A greedy algorithm for aligning DNA sequences. J Comput Biol 7:203–214 10890397 10.1089/10665270050081478

