# Supplementary material for: Investigating soil and ATCC bacterial strains for their ability to synthesize anisotropic gold nanoparticles
Source: Appl Microbiol Biotechnol. 2026 Jan 12;110:22. doi: 10.1007/s00253-025-13689-7 (PMC12799632; doi:10.1007/s00253-025-13689-7)
Supplement: Supplementary file 1 [file 253_2025_13689_MOESM1_ESM.docx]

**Supplementary Information**

**Investigating soil and ATCC bacterial strains for their ability to synthesize anisotropic gold nanoparticles**

Islam M. Ahmady^1,2^, Javad B. M. Parambath^3^, Elsiddig A. E. Elsheikh^1^, Gwangmin Kim^4^, Changseok Han^5,6^, Alejandro Pérez García^2^, Ahmed A. Mohamed^3^

Islam M. Ahmady	ORCID 0000-0003-0241-148X	(ialtayeb@sharjah.ac.ae)

Javad B. M. Parambath	ORCID 0000-0002-5069-2143	(jbasil@sharjah.ac.ae)

Elsiddig A. E. Elsheikh 	ORICD: 0000-0002-2949-5567	(eelsheikh@sharjah.ac.ae)

Gwangmin Kim	ORCID 0000-0003-0653-222X	(rhkdals0731@gmail.com)

Changseok Han	ORCID 0000-0002-8636-2859	(hanck@inha.ac.kr)

Alejandro Pérez García	ORCID 0000-0002-1065-0360	(aperez@uma.es)

Ahmed A. Mohamed	ORCID 0000-0001-7369-3117	(amohamed61@gmail.com)

^1^Department of Applied Biology, College of Sciences, University of Sharjah, Sharjah 27272, United Arab Emirates

^2^Departamento de Microbiología, Universidad de Málaga, and Instituto de Hortofruticultura Subtropical y Mediterránea “La Mayora” (IHSM-UMA-CSIC), 29071 Málaga, Spain

^3^Center for Advanced Materials Research, Research Institute of Sciences and Engineering, University of Sharjah, Sharjah 27272, United Arab Emirates

^4^Department of Civil and Environmental Engineering, Texas A&M University, College Station, Texas 77843, United States

^5^Program in Environmental and Polymer Engineering, Graduate School of INHA University, 100 Inha-ro, Michuhol-gu, Incheon 22212, Korea

^6^Department of Environmental Engineering, INHA University, 100 Inha-ro, Michuhol-gu, Incheon 22212, Korea

*Co-corresponding authors*

Alejandro Pérez García

Ahmed A. Mohamed

**Fig. S1** Antibacterial activities of the eight isolated *Streptomyces* spp. against a1-2. *E. coli* and b1-2. *S. aureus* showing zones of inhibition produced by strains 11, 28, and 36 against *S. aureus* but not on *E. coli*

**Table S1.** Zone of inhibition diameter for the eight isolated *Streptomyces* spp. ± standard deviation of 3 replicas

| ***Streptomyces* spp. isolate #** | ***E. coli*** | ***S. aureus*** |
| --- | --- | --- |
| **5** | - | - |
| **10** | - | - |
| **11** | - | 10.0 ± 0.3 mm |
| **28** | - | 11.0 ± 0.6 mm |
| **29** | - | - |
| **30** | - | - |
| **31** | - | - |
| **36** | - | 14.0 ± 0.6 mm |

**Fig. S2** Representative images of VITEK 2 identification results. a. strain 24 is *Elizabethkingia meningoseptica*, b. strain 26 is *Pseudomonas aeruginosa/**Pseudomonas fluorescens*, c. strain 33 is *Comamonas testosteroni,* and d. strain 35 is *Enterococcus casseliflavus*

**Fig. S3** Agarose gel electrophoresis images of *Streptomyces* spp. isolates used for 16S rRNA identification. a. Genomic DNA visualized on a 1% agarose gel. Lane M: 1 kb DNA ladder; Lanes 1–3: genomic DNA of strains 11, 28, and 36, respectively. b. PCR amplification of 16S rRNA gene products visualized on a 1.5% agarose gel. Lane M: 1 kb DNA ladder; Lanes 1–3: amplified products from strains 11, 28, and 36. Bands of approximately 560 bp and 700–800 bp confirm successful amplification of the partial 16S rRNA gene from the isolates

**Fig. S4** Phylogenetic tree. The tree shows the evolutionary relationships of 22 taxa based on partial 16S rRNA genes. It includes the isolates identified with 16S rRNA (in red boxes), their closest GenBank matches, Vitek-identified strains (in a blue box added using their GenBank reference sequences), and other reference strains, with *Desulfurococcus mobilis* set as the outgroup. The Neighbor-Joining bootstrap tree (1,000 replicates) is shown, with only well-supported branches retained. Distances were calculated using the Maximum Composite Likelihood method, resulting in 1,652 aligned positions. The analysis was completed in MEGA12.

**Fig. S5** Effect of incubation temperature on the synthesis duration of AuNPs at 28 °C and 37 °C for Gram-positive (right) and Gram-negative (left) bacteria

**Fig. S6** UV-vis spectrum of AuNPs synthesized from aryldiazonium salt by Gram-positive bacteria. a. *Streptomyces* sp. 11*,* b. *Streptomyces* sp. 28, c. *Dietzia sp.*, d. *E. faecalis* ATCC, e. *S. aureus* ATCC*,* and f. *L. acidophilus* ATCC*.* Bacterial cell suspensions of 6.0 × 10^8^ CFU/mL were incubated with 0.5 mM DS-AuCl_4_ at 28 °C and 37 ^o^C for 24 and 48 hours*.* Inset: images of the test tubes displaying the development of color

**Fig. S7** UV-vis spectrum of AuNPs synthesized from aryldiazonium salt by Gram-negative bacteria. a. *Pseudomonas* sp. 25*,* b. *Pseudomonas* sp. 26*,* c. *Pseudomonas* sp. 27*,* d. *Aeromonas* sp., e. *E. coli* ATCC, and f. *R. palustris* ATCC*.* Bacterial cell suspensions of 6.0 × 10^8^ CFU/mL were incubated with 0.5 mM DS-AuCl_4_ at 28 °C and 37 ^o^C for 24 and 48 hours*.* Inset: images of the test tubes displaying the development of color

**Fig. S8** EDS point analysis images of AuNPs synthesized by Gram-positive bacteria. a. *Streptomyces* sp. 11, b. *Streptomyces* sp. 28, *Dietzia* sp., d. *Enterococcus* sp., e. *E. faecalis* ATCC, f. *S. aureus* ATCC, and g. *L. acidophilus* ATCC

**Fig. S9** EDS point analysis images of AuNPs synthesized by Gram-negative bacteria a. *Elizabethkingia* sp., b. *Pseudomonas* sp. 26, c. *Pseudomonas* sp. 27, d. *Pseudomonas* sp. 25, e. *C.* *Comamonas* sp., f. *Aeromonas* sp., g. *S. algae* ATCC*,* h. *E. coli* ATCC, and i. *R. palustris* ATCC

**Fig. S10** TEM images with EDS mapping analysis of synthesized AuNPs under bacteria *Streptomyces* sp. 36
